# Editorial Comment: Comparison of Immediate vs Deferred Cytoreductive Nephrectomy in Patients With Synchronous Metastatic Renal Cell Carcinoma Receiving Sunitinib: The SURTIME Randomized Clinical Trial

**DOI:** 10.1590/S1677-5538.IBJU.2020.03.12

**Published:** 2020-02-20

**Authors:** A Bex, P Mulders, M Jewett, J Wagstaff, JV van Thienen, CU Blank, Stênio de C. Zequi

**Affiliations:** 1 The Netherlands Cancer Institute Amsterdam Netherlands The Netherlands Cancer Institute, Amsterdam, the Netherlands; 2 Radboud University Hospital Department of Urology Nijmegen Netherlands Department of Urology, Radboud University Hospital, Nijmegen, the Netherlands; 3 Princess Margaret Hospital Department of Urology TorontoOntario Canada Department of Urology, Princess Margaret Hospital, Toronto, Ontario, Canada; 4 Cardiff Hospital Department of Oncology Wales United Kingdom Department of Oncology, Cardiff Hospital, Wales, United Kingdom; 1 Fundação Prudente A. C. Camargo Cancer Center São PauloSP Brasil A. C. Camargo Cancer Center, Fundação Prudente, São Paulo, SP, Brasil

## COMMENT

Authors presented data from a waited prospective study, SURTIME, which evaluated the best time of cytoreductive nephrectomy (CN)and target therapy with sunitinib in two groups: CN performed before sunitinib versus Upfront CN followed by the therapy. Despite its low number of recruited patients (99) due a poor accrual (it was planned for 458 patients), authors found that the progression free survival at 28 weeks in intention to treat analysis, was similar in both groups. However, the overall survival was superior in patients on deferred surgical arm. This study was important in verify that the use of sunitinib before surgery can helps in identification of patients resistant to this drug and that probably will be not benefited with the surgery. On the other hand, a patients with satisfactory response to sunitinib can be maintained as suitable surgical candidates. Additionally, the patients on deferred CN arm received more frequently the drug in comparison the group of immediate nephrectomies. Although the results can influence our therapeutic decisions, caution in necessary: We must not to extrapolate these results for patients with no clear cell hystologies of kidney cancer, or for patients presenting with poor performance status or with central nervous system metastasis, since they were exclude from the SURTIME population. We do not know if the future, these findings will be replaced in similar way on trials that are using modern immunotherapy or using immunotherapy in combination with target therapies before or after cytoreduction. Let's wait for this new data.

